# Liver Fat Fraction and Machine Learning Improve Steatohepatitis Diagnosis in Liver Transplant Patients

**DOI:** 10.1002/nbm.70077

**Published:** 2025-06-09

**Authors:** Milan Hajek, Petr Sedivy, Martin Burian, Irena Mikova, Pavel Trunecka, Dita Pajuelo, Monika Dezortova

**Affiliations:** ^1^ MR Unit, Department of Diagnostic and Interventional Radiology Institute for Clinical and Experimental Medicine Prague Czech Republic; ^2^ Department of Hepatogastroenterology Institute for Clinical and Experimental Medicine Prague Czech Republic

**Keywords:** biomarkers, decision trees, liver fat, liver transplantation, machine learning, magnetic resonance spectroscopy, metabolic dysfunction‐associated steatohepatitis, metabolic syndrome

## Abstract

This study investigates the diagnostic accuracy of liver fat fraction (FF) and other biomarkers in differentiating metabolic dysfunction‐associated steatohepatitis (MASH) from non‐MASH conditions in a cohort of 127 liver transplant patients using ^1^H MRS and machine learning techniques. Receiver operating characteristic analysis identified FF as the most significant predictor, achieving an area under the curve (AUC) > 0.96 for distinguishing MASH from non‐steatosis and non‐MASH metabolic dysfunction‐associated steatotic liver disease (MASLD). Secondary biomarkers, including insulinemia and elastography, showed moderate discriminatory power (AUC = 0.7–0.8) and contributed to refining classification decisions within a decision tree model. The decision tree analysis, validated with 10‐fold cross‐validation and independent testing, demonstrated robust sensitivity and specificity, with FF contributing 60%–70% to decision‐making. Secondary splits, such as insulinemia (~16.21 μIU/mL) and elastography (~8 kPa), provided additional discriminatory power, particularly in cases with borderline FF values. Non‐significant biomarkers, such as waist circumference and signals of diallylic protons resonating at 2.8 ppm, were excluded due to low discriminatory performance (AUC < 0.7). Compared to the general population (~5.8% prevalence), MASH was significantly more common in liver transplant recipients (~30%–50%). In patients with FF > 5.3%, the positive predictive value (PPV) for MASH ranged from 88% to 97%, more than twice the PPV observed in the general population (approximately 60%). These findings align with existing literature validating MRI‐derived proton density fat fraction as a reliable biomarker for hepatic steatosis. However, liver fat percentage alone is insufficient for MASH diagnosis. Secondary biomarkers, particularly insulinemia and elastography, enhanced classification accuracy near the FF threshold of 5.3%. This multiparametric approach significantly improves diagnostic accuracy and addresses the elevated risk and unique clinical needs of liver transplant recipients. Overall, these results underscore the clinical utility and precision of MR spectroscopy as a noninvasive biomarker for MASH diagnosis in liver transplant patients.

Machine learning identifies liver fat fraction (FF) measured by ^1^H MR spectroscopy, insulinemia, and elastography as robust, non‐invasive biomarkers for diagnosing steatohepatitis in liver transplant patients, validated through decision tree analysis. Compared to the general population (~5.8% prevalence), MASH is significantly more common in liver transplant recipients (~30%–50%). In patients with FF > 5.3%, the positive predictive value for MASH ranged up to 97%, more than twice the value observed in the general population.

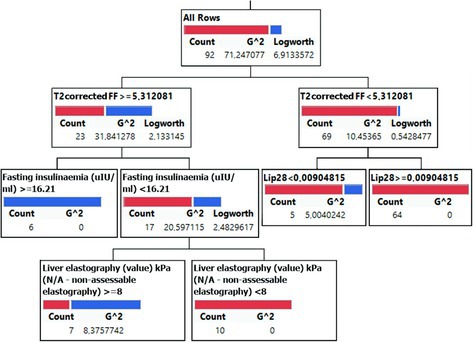

**Abbreviations**
AI: artificial intelligence
ALT: alanine aminotransferase
AST: aspartate aminotransferase
AUC: area under the receiver operating characteristic curve
BMI: body mass index
FF: fat fraction
f_SI_, f_UI_, f_PUI_
: fractions of hydrogen atoms in saturated, unsaturated, and polyunsaturated bonds
GGT: γ‐glutamyl transferase
HbA1c: glycated hemoglobin
HDL: high‐density lipoprotein
LDL: low‐density lipoprotein
MASH: metabolic dysfunction‐associated steatohepatitis
MASLD: metabolic dysfunction‐associated steatotic liver disease
ML: machine learning
*N*
: number of subjects
NAFL: nonalcoholic fatty liver
NAFLD: nonalcoholic fatty liver disease
NAS NAFLD: activity score
NASH: nonalcoholic steatohepatitis
NoS: non‐steatosis
PDFF: proton density fat fraction
QUICKI: quantitative insulin sensitivity check index
ROC: receiver operating characteristic curve
US: ultrasonography

## 1 Introduction

The nomenclature used to describe steatotic liver disease (SLD) has evolved considerably in recent years and now encompasses a range of conditions with different etiologies. Recently, leading scientific and professional societies, including the European Association for the Study of the Liver (EASL) and the American Association for the Study of Liver Diseases (AASLD), endorsed a shift from the term non‐alcoholic fatty liver disease (NAFLD) to metabolic dysfunction‐associated steatotic liver disease (MASLD) and its progressive form, metabolic dysfunction‐associated steatohepatitis (MASH) [1]. This updated terminology better reflects the critical role of metabolic dysfunction in disease pathogenesis. While this updated terminology emphasizes the metabolic drivers of disease, much of the literature still refers to NAFLD and NASH. For clarity, this article will use the terms MASLD and MASH, while continuing to refer to NAFLD and NASH when discussing previous studies.

MASLD is now the leading cause of chronic liver disease in Western countries, affecting 25–30% of the world's population. Recent modeling predicts a continued increase in MASLD prevalence in the US population from 33.7% in 2020 to 41.4% by 2050, with MASH prevalence rising from 5.8% to 7.9% [2]. Among liver transplant recipients, the prevalence of MASH is significantly higher; according to a meta‐analysis, approximately 38% develop recurrent MASH and about 17% develop de novo MASH within 5 years post‐transplantation [3]. These figures underscore the heightened vulnerability of transplanted livers to steatotic liver disease, highlighting the critical importance of accurate diagnostic methods in this patient population.

MASH has become a significant indication for liver transplantation (LT) in many countries. While liver biopsy remains the gold standard for diagnosing MASH [4], its invasive nature, associated risks, and sampling variability require reliable non‐invasive diagnostic methods. MR‐based methods, including chemical‐shift encoded multi‐echo MRI for quantification of proton density fat fraction (PDFF) or fat fraction (FF) calculation from ^1^H MRS, are an integral part of routine clinical assessment and provide rapid, reliable, and non‐invasive measurements of liver fat content [5, 6, 7, 8, 9, 10, 11, 12, 13]. These methods correlate strongly with the histologic grading of steatosis [14] and are increasingly utilized in clinical trials to monitor NAFLD activity score (NAS) and related histologic outcomes. However, while FF or PDFF effectively quantifies liver fat and identifies steatosis severity, these parameters show limited correlation with other critical NAS components essential for definitive MASH diagnosis, such as lobular inflammation, hepatocellular ballooning, and fibrosis [15, 16]. This limitation underscores the necessity for additional non‐invasive biomarkers or imaging parameters to comprehensively assess the full spectrum of liver pathology in MASH. This gap reflects the absence of simple, liver‐specific, non‐invasive biomarkers that adequately capture the full spectrum of NAS features. Addressing this challenge opens the door to enhancing diagnostic precision by applying multiparametric and multimodal diagnostic approaches, including quantitative imaging techniques (e.g., MR‐based methods, ultrasound techniques namely attenuation imaging, controlled attenuation parameter, or ultrasound elastography‐based techniques) combined with serum biomarkers or computational models.

Machine learning (ML) methods have emerged as promising tools for integrating and analyzing multimodal data to improve diagnostic accuracy. Techniques, namely decision trees, neural networks, and random forests, excel in combining clinical, laboratory, and imaging variables to create robust diagnostic models [17, 18, 19, 20, 21]. In MASLD and MASH, ML has successfully identified important predictors such as BMI, liver enzymes, and MR‐based fat measurements. Recent studies demonstrate that ML models can differentiate MASLD from MASH and predict advanced MASH and fibrosis with high sensitivity and specificity based on routinely available clinical information [22]. Despite challenges related to data standardization and model interpretability, ML represents a transformative approach to MASLD/MASH diagnostics [23].

However, it is essential to assess whether the added complexity of ML methods provides real clinical benefits over simpler approaches. Comparing ROC‐based imaging biomarkers (e.g., MR‐derived fat fraction) with ML models can help determine if additional variables meaningfully enhance diagnostic accuracy or patient characterization.

In a previous pilot study [24], we found that ^1^H MR spectroscopy of the liver can identify post‐transplant patients with high FF values corresponding to NAFLD or even NASH (now MASLD and MASH). This study aims to validate the hypothesis that increased liver fat content, described by FF and other laboratory and clinical biomarkers, correlates with diagnosing MASH in post‐LT patients. Additionally, it examines whether MR‐measured fat content, in combination with other parameters, can serve as a biomarker for MASH in liver transplant recipients, using ROC analysis and decision tree ML methods.

## 2 Patients and Methods

### 2.1 Subjects

We conducted 231 clinical, MRI, MRS, and laboratory examinations on 135 liver transplant recipients living between 6 months and 20.6 years after their liver transplant (LT) between 2015 and 2020. Biopsy of the liver graft was initially performed 1 year after LT according to the local protocol (within ± 24 h of the MR examination). Of the total 231 examinations, biopsy results were obtained in 162 examinations. In 35 patients, multiple biopsies were performed. For the next data analysis step, we formed a group of 127 subjects with only one biopsy. The data from this group were divided into three subgroups based on the histopathologic NAFLD activity score (NAS) from the liver biopsy:

NoS (non‐steatosis) patients: NAS ≤ 2

MASLD patients: NAS 3–4 points

MASH patients: NAS ≥ 5 points (7 subjects with steatosis S2 and 10 with steatosis S3)

Shear wave elastography was routinely performed using SuperSonic Imagine Aixplorer (SuperSonic Imagine, France), and the resulting values were treated as continuous variables. An overview of the basic clinical and laboratory data of the patients in the individual histology subgroups can be found in Table 1.

**TABLE 1:** Clinical, laboratory, and MRS results of 127 studied subjects.

	NoS (NAS ≤ 2)	MASLD (NAS 3–4)	MASH (NAS ≥ 5)
Number of subjects	90	20	17
	Median (25th–75th percentile)	Median (25th–75th percentile)	Median (25th–75th percentile)
Age (years)	57.55 (46.40–66.45)	58.90 (45.82–65.76)	63.50 (51.30–68.58)
Post‐transplantation period (years)	1.14 (1.01–2.19)	1.96 (1.02–4.92)	1.22 (1.02–3.52)
Elastography (kPa)	6.50*** (5.90–7.40)	7.45 (6.45–9.40)	8.20 (7.65–10.25)
BMI (kg/m^2^)	25.25** (22.86–29.48)	26.95 (24.02–29.49)	29.94 (27.29–32.96)
Waist circumference (cm)	94.5*** (85.8–106.0)	100.5 (92.3–106.8)	107.0 (104.0–113.5)
Bilirubin (μmol/L)	12.50 (9.20–20.08)	12.45 (9.41–7.23)	13.20 (8.35–18.40)
AST (μkat/L)	0.38 (0.30–0.46)	0.40 (0.34–0.58)	0.41 (0.35–0.56)
ALT (μkat/L)	0.44* (0.35–0.56)	0.51 (0.38–0.64)	0.59 (0.45–0.76)
ALP (μkat/L)	1.45 (1.14–1.90)	1.33 (1.03–1.87)	1.69 (1.09–2.27)
GGT (μkat/L)	0.36* (0.26–0.61)	0.40 (0.31–0.71)	0.64 (0.34–1.44)
Total cholesterol (mmol/L)	4.55 (3.90–5.10)	4.95 (4.00–5.80)	5.10 (4.35–5.90)
LDL (mmol/L)	2.70 (2.25–3.05)	2.95 (2.23–3.28)	3.20 (2.20–3.50)
HDL (mmol/L)	1.31 (1.03–1.48)	1.05 (0.88–1.40)	1.11 (0.86–1.47)
Fasting blood glucose (mmol/L)	5.32** (4.97–6.17)	5.22 (4.72–7.53)	5.94 (5.59–7.15)
HbA1c (mmol/mol)	36* (32–40)	35 (33–46)	43.0 (34.5–48.0)
C‐peptide (nmol/L)	0.83** (0.61–1.14)	0.85* (0.70–1.13)	1.37 (1.09–1.54)
Fasting insulinemia (μIU/mL)	6.54** (4.37–10.48)	7.00 (4.60–10.31)	14.43 (8.80–20.63)
HOMA‐IR (mmol/L/22.5)	1.55** (1.08–2.61)	1.65* (1.08–3.26)	4.20 (2.19–6.06)
QUICKI (mg/dL)	0.36** (0.33–0.38)	0.35* (0.32–0.38)	0.31 (0.30–0.34)
FF	1.10***^,+^ (0.80–1.83)	4.01* (2.29–9.55)	14.81^+^ (9.38–25.31)
f_SI_	0.83**** (0.78–0.87)	0.88 (0.85–0.90)	0.89 (0.87–0.90)
f_UI_	0.058****^,++^ (0.041–0.090)	0.042 (0.029–0.049)	0.038 (0.018–0.042)
f_PUI_	0.022**** (0.015–0.045)	0.015 (0.009–0.024)	0.010 (0.007–0.018)

Abbreviations: ALT, alanine aminotransferase; AST, aspartate aminotransferase; BMI, body mass index; FF, liver fat fraction; f_PUI_, fractions of hydrogen atoms in polyunsaturated bonds; f_SI_, fractions of hydrogen atoms in saturated bonds; f_UI_, fractions of hydrogen atoms in unsaturated bonds; GGT, γ‐glutamyl transferase; HbA1c, glycated hemoglobin; HDL, high‐density lipids; HOMA‐IR, homeostatic model assessment of insulin resistance; LDL, low‐density lipoprotein; QUICKI, quantitative insulin sensitivity check index.

Significant differences from MASH: *p < 0.05; **p < 0.005; ***p < 0.0005; ****p < 0001; significant differences from MASLD: ^*+*^ *p* < 0.05; ^*++*^ *p* < 0.01.

The experimental protocol was approved by the local ethics committee and conducted according to the Declaration of Helsinki. All subjects gave their written informed consent before participating in the study.

### 2.2 MR Spectroscopy

The MR examinations were performed on a 3‐T MR whole‐body system (MAGNETOM Trio, Siemens Healthineers, Erlangen, Germany) with the patient in the supine position. The system was equipped with an eight‐channel surface body and spine array coils.

MR images were acquired in held expirations with standard localizer and half‐Fourier acquisition single‐shot turbo spin‐echo (HASTE) sequences in both the transverse and coronal directions (repetition time [TR]/echo time [TE] was 1800/96 ms, 20 slices, slice thickness 10 mm, in‐plane resolution 1.56 × 1.56 mm).

^1^H MRS was performed using a point‐resolved spectroscopy sequence (PRESS: TR/TE = 4500/30 ms, 1 acquisition). A volume of interest (VOI) of 40 × 30 × 25 mm^3^ was placed near the biopsy position in the V/VIII liver segment. Single spectra in a held expiration were repeated three times without water suppression, and water‐suppressed spectra (50 Hz bandwidth) were measured twice. In addition, a set of PRESS spectra with TEs of 30, 50, 68, 135, 180, and 270 ms was acquired without water suppression to estimate T_2_ relaxation times. Nevertheless, due to the unacceptable variance of the mono‐exponential model, we finally used values from our previous studies (T_2_ water = 27 ms, T_2_ lipids = 60 ms) [12].

After rigorous quality control tests, we selected 1110 non‐water‐suppressed spectra and 740 water‐suppressed spectra for subsequent data analysis using LCModel [25]. The basis set included water (4.7 ppm) and 10 lipid signals (Lip) resonating at 0.9 ppm (terminal methyl groups in long chain alkanes; ‐(CH2)n‐**CH3**), 1.3 ppm (methylene groups; ‐(**CH2**)n‐), 1.6 ppm (β**‐**methylene in fatty acids‐CO‐CH2‐**CH2**‐), 2.1 ppm (allylic methylene; ‐CH2‐**CH=CH**‐CH2‐), 2.4 ppm (α‐methylene; ‐CO‐**CH2**‐CH‐), 2.8 ppm (diallylic methylene; ‐CH=CH‐**CH2**‐CH=CH‐), 4.2 ppm, 4.4 ppm (ester groups; ‐**CH2**‐O‐CO‐, and 5.3 ppm (vinylic groups; **CH=CH**‐) with 5.2 ppm contribution. The signals at 4.2 and 4.4 ppm were excluded from further analysis. The characteristic spectrum is shown as a part of Figure 2. The fat fraction (FF) was calculated from the T_2_‐corrected fatty acid (FA) chain intensities (I^cor^_F_) and corrected water signal intensity (I^cor^_W_), including the signal at 5.3 ppm.

(1) $$\mathrm{FF}\left[\%\right]=100\times \frac{I_{F}^{\mathrm{cor}}}{I_{F}^{\mathrm{cor}}+I_{W}^{\mathrm{cor}}}$$

Fractions of hydrogen atoms in saturated (f_SI_: ‐CH_2_‐, ‐CH_3_), unsaturated (f_UI_: ‐CH=CH‐), and polyunsaturated (f_PUI_: (‐CH=CH‐)_n_) bonds in all FA chains were calculated using the following equations and were used to construct FA spectral profiles [24, 26]:

(2) $$f_{\mathrm{SI}}=\frac{\left(\mathrm{Lip}09+\mathrm{Lip}13+\mathrm{Lip}16+\mathrm{Lip}24\right)}{\left(\mathrm{Lip}09+\mathrm{Lip}13+\mathrm{Lip}16+\mathrm{Lip}21+\mathrm{Lip}24+\mathrm{Lip}28+\mathrm{Lip}53\right)}$$

(3) $$f_{\mathrm{UI}}=\frac{\left(\mathrm{Lip}21/2+\mathrm{Lip}28\right)\;}{\left(\mathrm{Lip}09+\mathrm{Lip}13+\mathrm{Lip}16+\mathrm{Lip}21+\mathrm{Lip}24+\mathrm{Lip}28+\mathrm{Lip}53\right)}$$

(4) $$f_{\mathrm{PUI}}=\frac{\left(\mathrm{Lip}28\right)}{\left(\mathrm{Lip}09+\mathrm{Lip}13+\mathrm{Lip}16+\mathrm{Lip}21+\mathrm{Lip}24+\mathrm{Lip}28+\mathrm{Lip}53\right)}$$

### 2.3 Statistics

Data management was performed using Microsoft Excel. Statistical analyses, including chi‐square tests, *t*‐tests, and Mann–Whitney *U*‐tests, were conducted using GraphPad Prism 10 software. For parametric data, one‐way ANOVA with the Holm–Sidak multiple comparison test was used, while for non‐parametric data, the Kruskal–Wallis test with the Dunn multiple comparison test was used.

**FIGURE 1:**
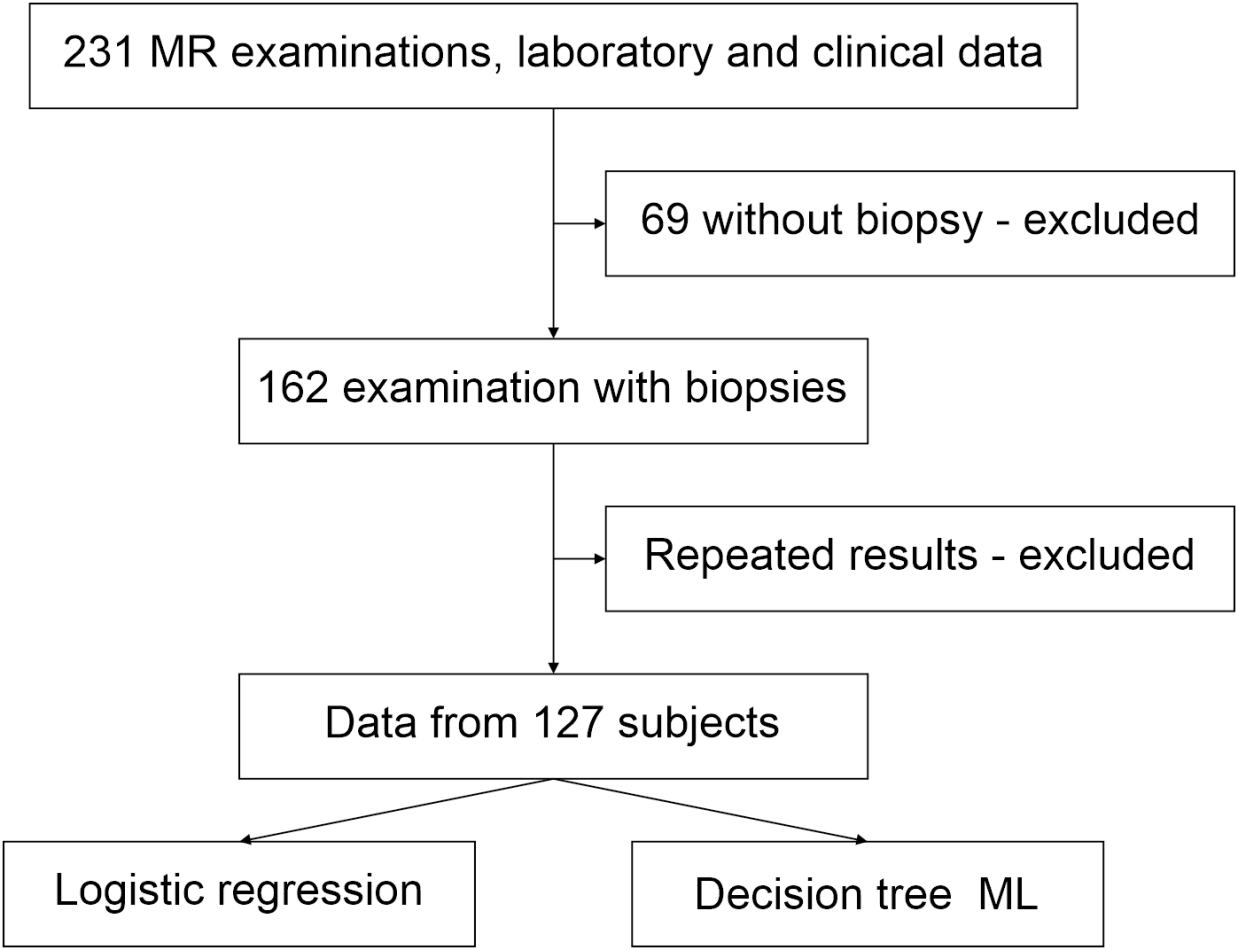
From a total of 231 MR examinations and laboratory data, 127 individual examinations from single patients with histologic results were used as a dataset for training, validation, and prediction using machine learning.

Logistic regression (stepwise analysis) and decision tree machine learning methods were used for classification and regression tasks using the Fit Model platform of the JMP 17.2 statistical software. To test the hypothesis that FF is an essential marker for MASH, patients were categorized into two groups based on NAS score: those with MASH and those without MASH (i.e., the NoS and MASLD groups). The data flow is shown in Figure 1.

Performance metrics, including AUC, sensitivity, specificity, accuracy, precision, and PPV (Positive Predictive Value), were calculated using GraphPad Prism. Decision tree analyses were conducted using JMP 17.2 software, with the significance of predictor variables assessed using G^2^ (Pearson chi‐squared statistic) and LogWorth values. LogWorth is defined as the negative base‐10 logarithm of the *p*‐value (LogWorth = −log_10_(*p*‐value)). In this study, splits with LogWorth values below 1.3 (*p*‐value > 0.05) were considered statistically insignificant and excluded from further interpretation to ensure robust model performance. This threshold balances the need for statistical rigor and minimizing false positive rates. For all other analyses, a *p*‐value < 0.05 was considered statistically significant.

## 3 Results

### 3.1 Lipid Profile

**FIGURE 2:**
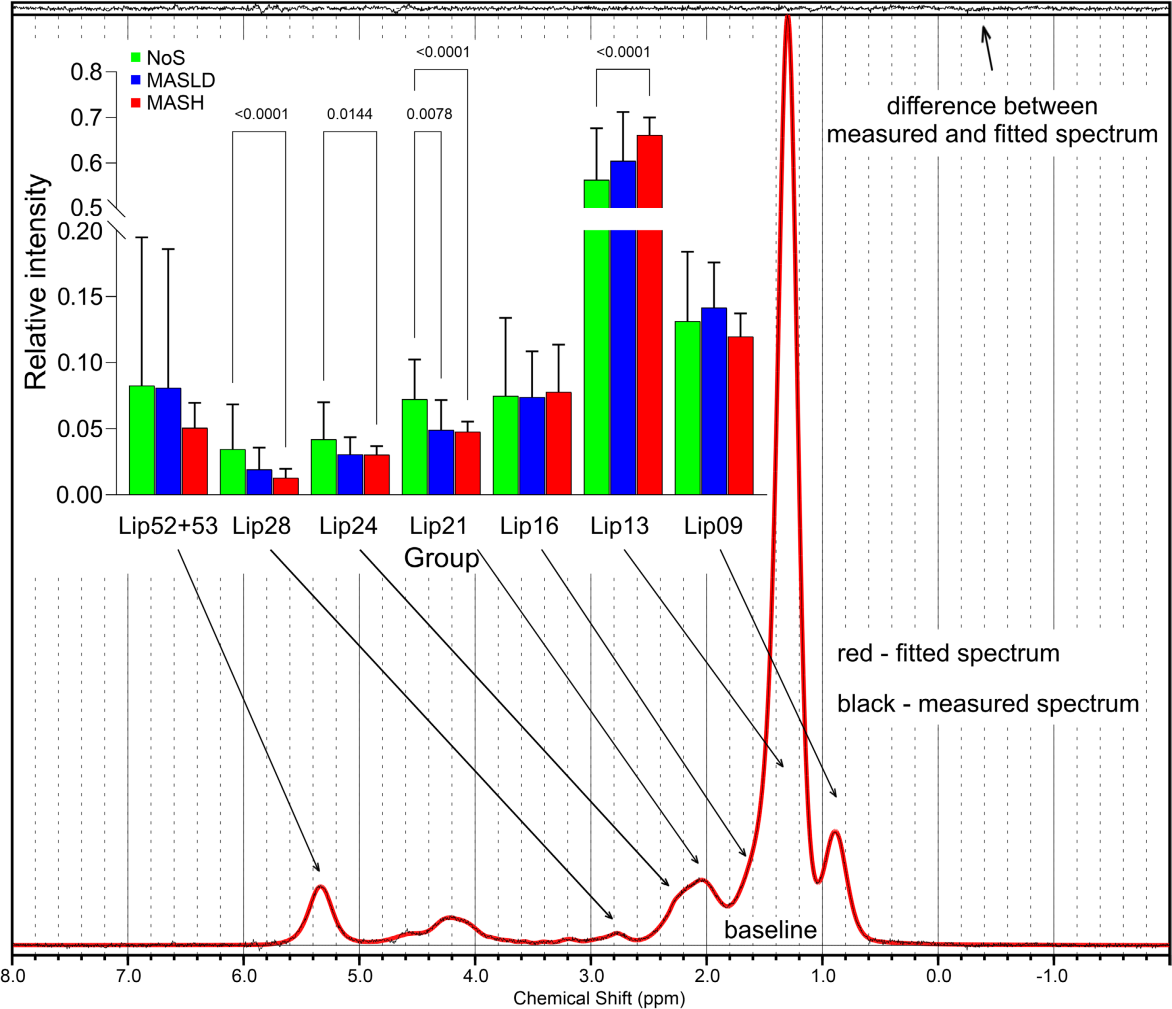
LCModel output of ^1^H MR spectrum with lipid signals assessment together with mean relative signal intensities and their standard deviations in the groups of patients with no steatosis (NoS), MASLD, and MASH.

^1^H MR liver spectra from all 127 patients in the NoS, MASLD, and MASH groups were compared. Multi‐parametric comparison using ANOVA revealed significant differences between the NoS and MASH groups in the relative intensities of aliphatic CH_2_ signals (Lip13, Lip24), allylic (Lip21) signals, and diallylic (Lip28) signals. In addition, the relative intensities of Lip21 even distinguished the MASLD group from the NoS group (see Figure 2).

These trends in lipid profiles suggest a loss of double‐bonded fatty acids with increasing fat content in the liver. The derived parameters f_SI_, f_UI_, and f_PUI_ showed significant differences among the NoS, MASLD, and MASH groups (see Table 1). This finding aligns with our observations across steatosis grades S0–S3 and supports conclusions from previous studies and referenced literature [24, 26].

### 3.2 Analysis of the Receiver Operating Characteristic Curve (ROC)

The area under the ROC curve (AUC), which plots sensitivity against (1 − specificity), was calculated to determine the ability of each parameter to distinguish MASH from the NoS + MASLD group. The values closer to 1 indicate excellent discriminatory ability of the feature.

**FIGURE 3:**
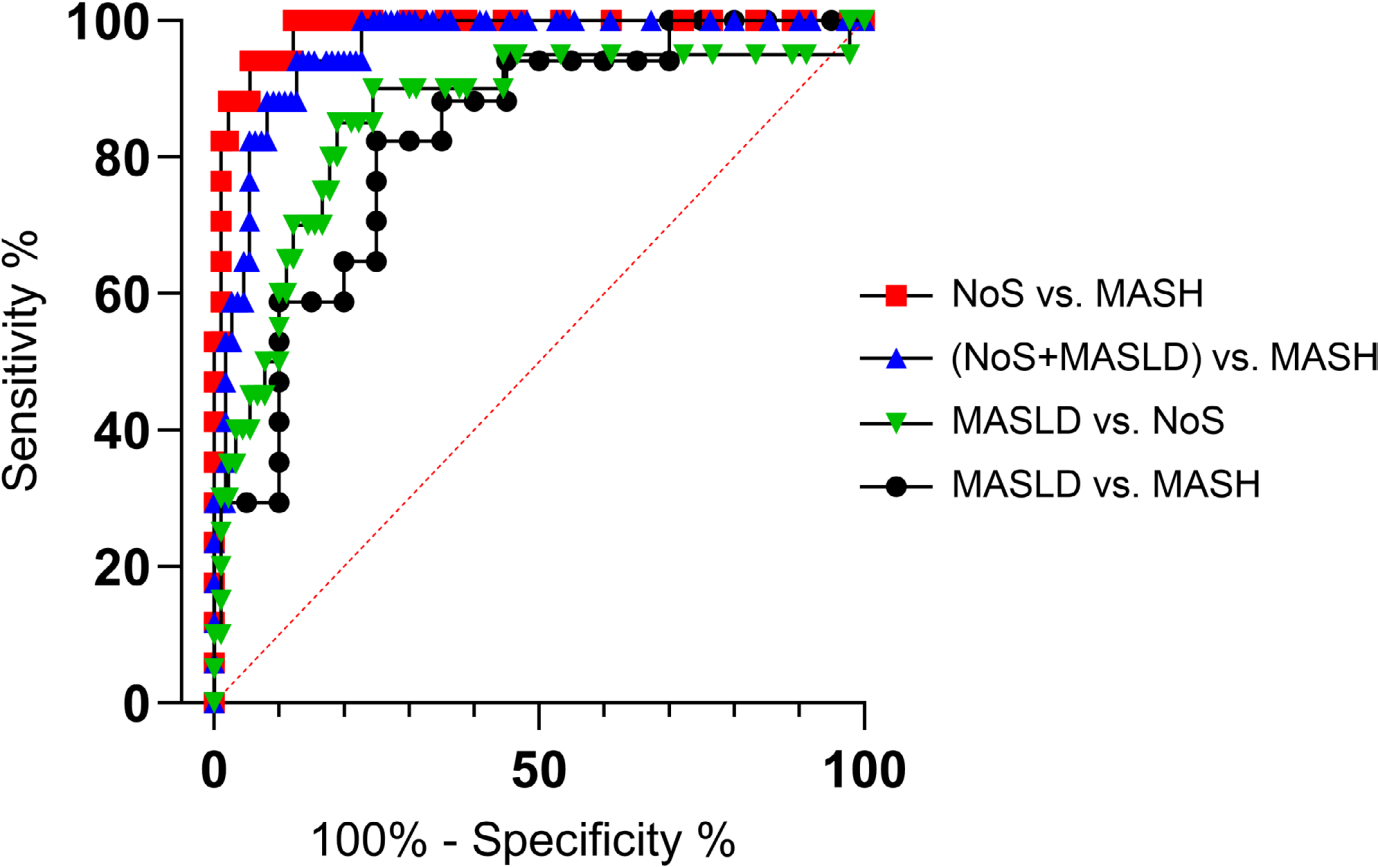
ROC curves for FF are based on the following group comparisons. Excellent discriminatory ability was observed for the MASH versus NoS + MASLD (AUC = 0.9561) and MASH versus NoS (AUC = 0.9850) groups. A good discriminatory ability was calculated for the groups MASH versus MASLD (AUC = 0.8265) and MASLD versus NoS (AUC = 0.8567).

The results, summarized in Table 2, include sensitivity (fraction of NASH subjects correctly identified as positive), specificity (fraction of NoS + MASLD subjects correctly identified as negative), Youden's index, and cut‐off points for 22 clinical and laboratory variables. The FF parameter demonstrated the highest AUC value, indicating superior discriminatory ability. Individual biomarkers, such as insulinemia and the quantitative insulin sensitivity check index (QUICKI), influenced by insulin levels, exhibited comparable discriminatory power with AUC values between 0.7 and 0.8. In addition, anthropometric measures such as waist circumference and BMI showed a high sensitivity, which is consistent with the existing literature. Other MR parameters also showed considerable AUC values, further contributing to the differentiation between MASH and non‐MASH groups. Variables with AUC values < 0.7, marked in italics in Table 2, were excluded from further analysis due to their poor discriminatory ability. Figure 3 illustrates the graphical results of ROC analysis for FF, comparing the following subject groups: MASH versus NoS, MASH versus MASLD, MASH versus (NoS + MASLD), and NoS versus MASLD.

**TABLE 2:** The AUC, sensitivity, specificity, and cut‐off points of the ROC analysis were calculated for 22 clinical and laboratory data parameters for the MASH versus (NoS + MASLD) groups.

Variable	AUC	Cut‐off value	Sensitivity%	Specificity%	Youden index
FF	0.9561	> 5.206	94.12	87.27	81.39
f_SI_	0.8000	> 0.8417	100	51.82	51.82
Lip13	0.7842	> 0.6118	100	52.73	52.73
QUICKI	0.7784	< 0.3145	62.5	91.67	54.17
f_UI_	0.7690	< 0.04270	88.24	68.18	56.40
Waist circumference (cm)	0.7663	> 102.5	82.35	69.09	51.44
Elastography (kPa)	0.7609	> 7.850	76.47	82.00	58.47
Fasting insulinemia (μIU/mL)	0.7598	> 8.435	81.25	66.67	47.92
Lip21	0.7596	< 0.05996	100	53.64	53.64
C‐peptide (nmol/L)	0.7554	> 1.065	81.25	70.64	51.89
BMI	0.7545	> 26.30	88.24	57.01	45.25
Lip28 = f_PUI_	0.7471	< 0.02108	94.12	52.73	46.85
Triglycerides (mmol/L)	0.7439	> 1.295	88.24	55.45	43.69
Fasting glycemia (mmol/L)	0.7273	> 5.410	100	56.36	56.36
*ALT (μkat/L)*	*0.6842*	*> 0.5750*	*64.71*	*76.36*	*41.07*
*GGT (μkat/L)*	*0.6810*	*> 0.4950*	*70.59*	*68.18*	*38.77*
*HbA1c (mmol/mol)*	*0.6781*	*> 42.50*	*52.94*	*85.45*	*38.39*
*Cholesterol (mmol/L)*	*0.6476*	*> 4.750*	*70.59*	*56.36*	*26.95*
*LDL (mmol/L)*	*0.6389*	*> 2.950*	*66.67*	*64.49*	*31.16*
*Lip52 + 53*	*0.6190*	*< 0.05859*	*88.24*	*49.09*	*37.33*
*AST (μkat/L)*	*0.6182*	*> 0.4650*	*47.06*	*76.36*	*23.42*
*ALP (μkat/L)*	*0.5655*	*> 1.685*	*52.94*	*64.55*	*17.49*

*Note:* An excellent discrimination was only found for the FF parameter. The parameters in italics were not used for further analysis due to poor discrimination (AUC values < 0.7).

Abbreviations: ALT, alanine aminotransferase; AST, aspartate aminotransferase; AUC, area under the receiver operating characteristic curve; BMI, body mass index; FF, liver fat fraction; f_SI_, f_UI_, f_PUI_, fractions of hydrogen atoms in saturated, unsaturated, and polyunsaturated bonds, resp.; GGT, γ‐glutamyltransferase; HbA1c, glycated hemoglobin; LDL, low‐density lipoprotein; Lip13, Lip21, Lip28, Lip52 + 53, relative intensities of all lipid signals, excluding glycerol signals; QUICKI, quantitative insulin sensitivity check index.

### 3.3 Decision Tree

Fourteen variables with the highest AUC from the ROC analysis were selected as features for the multiparametric decision tree machine learning method using the JMP software. Data from all 127 subjects were automatically divided into training and validation sets to determine the number of root nodes and leaves in the model.

A 10‐fold cross‐validation with validation proportions of 30% and 50% was performed to avoid overfitting. A 70:30 split of training and validation was chosen as the primary model as it has better metrics, including higher AUC and accuracy. Although the 50:50 split’s results were slightly worse on some metrics, they were consistent with the 70:30 model, confirming FF’s robustness as a significant predictor of MASH. Metrics such as sensitivity, specificity, accuracy, and positive predictive values confirmed that the decision tree model did not overfit, as the performance was consistent across the training and validation sets. Performance metrics for the decision tree results are presented in Table 3. Comparable values from the ROC analysis are also included in Table 3.

**TABLE 3:** Performance metrics of decision tree model (mean from 10‐fold cross‐validation) and ROC analysis at different prevalence levels.

Method	Sensitivity	Specificity	Accuracy	AUC	Cut‐off	Prevalence	PPV
Decision tree training	0.8185	0.984	0.9178	0.97	> 5.3	40%	97%
	0.8185	0.984	0.974	0.97		5.8%	75%
Decision tree validation	0.5443	0.961	0.784	0.89	> 5.3	40%	90%
	0.5443	0.961	0.936	0.89		5.8%	46%
Decision tree mean	0.6814	0.9725	0.8560	0.98	> 5.3	40%	94%
	0.6814	0.9725	0.9055	0.90		5.8%	60%
ROC	94.12	87.27	0.9002	0.96	> 5.206	40%	83%
	94.12	87.27	0.8769	0.96		5.8%	31%

*Note:* Sensitivity = TP/(TP + FN), Specificity = TN/(TN + FP), Estimated Accuracy = (Sensitivity × Prevalence) + (Specificity × (1 − Prevalence)), PPV = (Sensitivity × Prevalence)/[[(1 − Specificity) × (1 − Prevalence)] + (Sensitivity × Prevalence)].

Abbreviations: FN = false negatives; TP = true positives.

**FIGURE 4:**
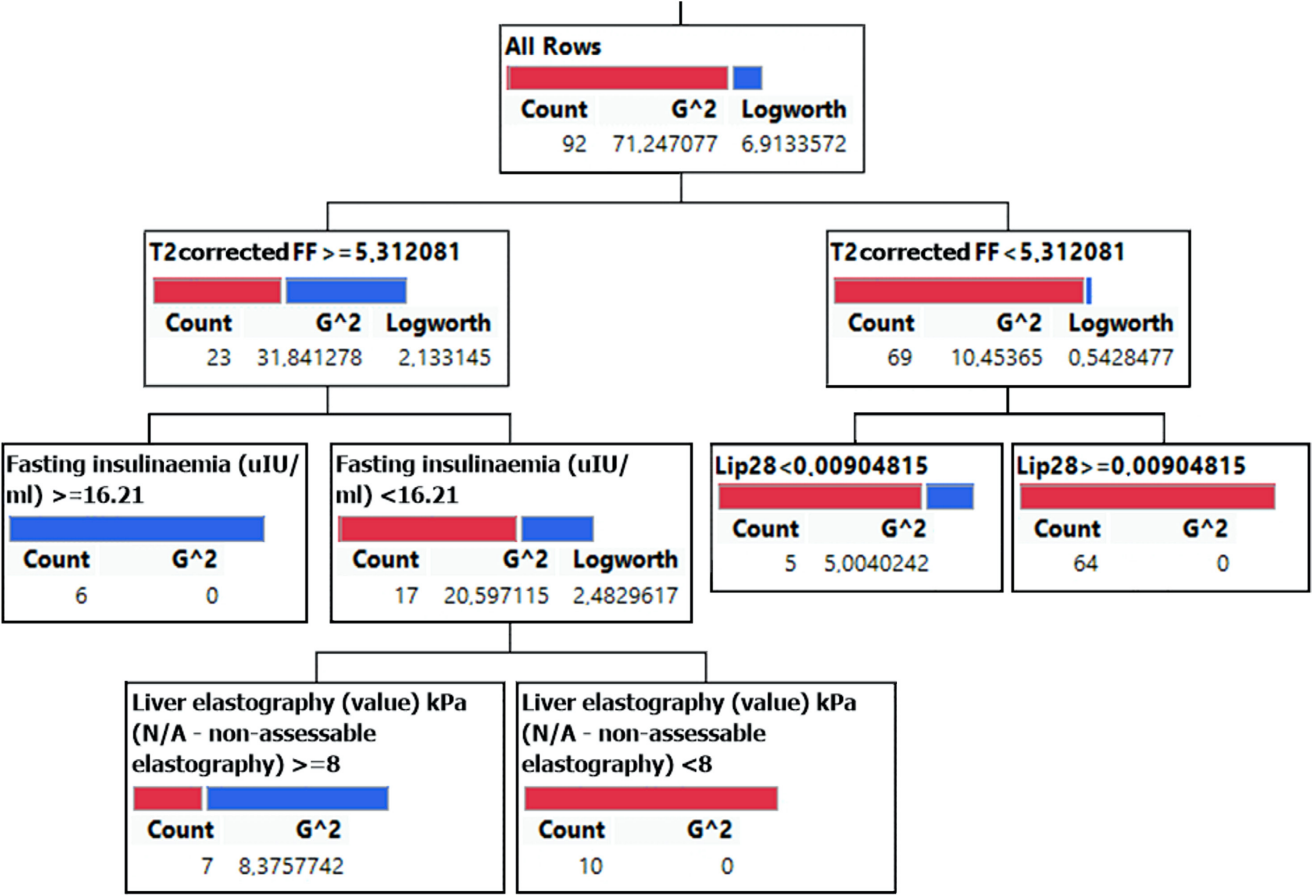
The decision tree model was derived from the 70:30 training‐validation split, which achieved high predictive accuracy through 10‐fold cross‐validation. Details are seen in the text.

Figure 4 depicts the decision tree structure for the 70:30 split. The model begins with FF as the root node (threshold = 5.31), followed by secondary splits based on insulinemia (~16.21 μIU/mL) and elastography (~8 kPa). Variables such as Lip28 (f_PUI_) and waist circumference showed minimal contribution and were excluded from interpretation.

The column contributions quantifying the importance of each feature revealed that FF was the dominant predictor, contributing 60%–70% to the decision‐making process. Secondary features, including insulinemia, elastography, PUI, and Lip28 (f_PUI_), contributed 15%–25% at the lower nodes.

The decision tree results were substantiated using a nominal logistic fit. Only FF and insulinemia had significant effects, with LogWorth values of 10.44 and 1.75 and *p*‐values < 0.017. The Wald test also confirmed that FF (*p* < 0.0001) and insulinemia (*p* < 0.0128) were significant predictors of MASH. Table 4 summarizes the ranges of cut‐offs for parameters identified as significant (LogWorth ≥ 1.3) or non‐significant (LogWorth < 1.3) in the decision tree model. Fat fraction (FF) consistently showed significance, while other parameters, like insulinemia and elastography, had variable contributions.

**TABLE 4:** Ranges of cut‐offs and significance levels of parameters from the decision tree model (70:30 split, 10‐fold cross‐validation).

Parameter	Cut‐offs	Significant splits (LogWorth ≥ 1.3)	Non‐significant splits (LogWorth < 1.3)
FF	~5.3%–6.7%	Always significant	None
Insulinemia	~16.21–17.22 μIU/mL	Frequently significant	Rare (low‐level splits)
Elastography	~7.4–8.1 kPa	Occasionally significant	Often non‐significant
C‐Peptide	~1.29 nmol/L	Sometimes significant	Sometimes non‐significant
*f*_*PUI*_	*~0.009*	*None*	*Always non‐significant*
*Waist circumference*	*~109 cm*	*None*	*Always non‐significant*

The decision tree model (validation sets 30% and 50%) was also tested in an independent cohort of 30 subjects, four of whom had histologically confirmed MASH. Sensitivity and specificity were calculated as 1/0.714 and 1/0.880 for FF cut‐off values of 5.6% and 7.2%, respectively. These results indicate an effective classification of non‐MASH patients in both cases, with a low number of false‐positive results (8 in the first model and 3 in the second model).

These results demonstrate the predictive power of FF as the main feature, with insulinemia and elastography contributing to the refinement of the classification.

## 4 Discussion

Our analysis of 127 MR examinations in liver transplant patients demonstrates that fat fraction (FF) values can effectively distinguish between non‐steatosis (NoS), non‐MASH (MASLD), and MASH groups with high sensitivity and specificity, utilizing ROC analysis, decision trees, and nominal logistic regression methods.

Probability calculations derived from decision‐tree analysis and ROC‐derived sensitivity and specificity values combined with prevalence estimates support FF's pivotal role as a primary biomarker in diagnosing MASH in liver transplant recipients. These underscore the diagnostic utility of MR methods in diverse patient populations.

The development of MASH in patients after liver transplantation is well‐documented, with prevalence rates reported between 30% and 50% due to recurrence or de novo development. According to the PPVs derived from the decision‐tree model for the 40% prevalence, the probability that post‐transplant patients suffer from MASH ranges from 90% to 97%, whereas ROC analysis estimates this probability at approximately 83%. These probabilities are roughly twice as high as those in the general population (PPVs of 31% to 60%), where the prevalence of MASH is approximately 5.8%, assuming a liver fat threshold of approximately 5.3%. These calculations highlight the clinical usefulness and diagnostic accuracy of MR measurements of hepatic fat content in liver transplant recipients.

FF consistently achieved optimal results among all variables tested using ROC analysis and the decision tree (AUC > 0.96). Secondary variables such as insulinemia and elastography also played an important role in refining the classification process within the decision tree model. These findings align with previous research highlighting the pivotal role of steatosis in liver disease progression. For instance, Middleton et al. [15] demonstrated that the majority of patients with nonalcoholic steatohepatitis (NASH) exhibit high‐grade steatosis. Their study found a strong agreement between MRI‐derived PDFF measurements and pathologist‐assigned steatosis grades, underscoring the efficacy of MR methods in assessing this NAFLD activity score (NAS) component.

Our decision tree model strikes an optimal balance between higher sensitivity and specificity and ensures robust classification of MASH and non‐MASH groups. This methodology highlights the clinical applicability of our classification system, particularly in liver transplant patients who benefit from noninvasive and precise diagnostic tools. Furthermore, insulinemia provided additional discriminatory power at the lower levels of the tree, especially in patients with FF values below the threshold of 5.3%. At the same time, elastography helped characterize liver stiffness in advanced stages.

Our results support the findings of Panagiotopoulos et al. who found that proton density fat fraction (PDFF) is a discriminator of NASH (now referred to as MASH) in a group of obese patients [27]. They are also consistent with the general findings of studies focusing on the treatment of NASH [6, 28, 29, 30] showing that a significant relative decrease in MRI‐PDFF (≥ 30% rel.) is associated with a greater histologic response in NASH, such as a decrease in NAS by about 2 points.

In clinical practice, the diagnosis of MASH often involves a combination of noninvasive imaging techniques, laboratory tests, clinical evaluation, and biopsy results. Efforts to improve the diagnosis of liver disease have recently focused on using multivariate statistical methods to process the results of noninvasive imaging, spectroscopy, and clinical and laboratory tests.

An analysis of extensive datasets has revealed a range of complex parameters that hold promise in identifying MASLD and various degrees of liver fibrosis [31]. In addition, animal studies [32] have highlighted the significant value of FF values when used in conjunction with MR elastography to characterize MASH [33]. Several research projects have investigated the utility of imaging techniques, particularly MRI and ultrasound, for diagnosing MASH in patients with MASLD and identifying the presence and stages of liver fibrosis.

Troelstra et al. [34] compared MRS, MRI, and US methods in a cohort of 37 subjects with histologically confirmed MASLD. They employed ROC analysis to assess the performance of MR and other imaging methods in distinguishing between steatosis and MASH, documenting AUC values ranging from 0.52 to 0.79.

Another study examined 737 subjects with MASLD identified using MRS. The authors used biomarkers to create the Dallas Steatosis Index [35], a computational tool for differentiating between subjects with and without MASLD (AUC 0.824). Neural network calculations based on large datasets obtained from electronic health records have provided information on various biochemical predictors, such as ALT, AST, and platelet count [36]. In a cohort comprising hundreds of thousands of subjects, the specificity and sensitivity of MASLD/MASH identification calculated using ROC AUC values were approximately 0.8 to 0.9 across the methods used.

On the other hand, it is known that better results can be achieved when the patient cohort is more homogeneous. This applies to our group of patients, all of whom underwent liver transplantation, received standardized post‐transplantation care in a single center, and were examined according to a standard protocol using biochemical tests and liver biopsy (starting one year after LT), in addition to MR determination of liver steatosis and profiles of fatty acids. Results based on the combination of FF and the 14 most significant clinical and laboratory biomarkers resulting from ROC analysis (AUC > 0.7) correspond to the study by Okanoue et al. [19]. They used a machine learning method called NASH‐Scope to characterize MASLD and MASH based on 11 biochemical parameters, enrolling 324 and 74 patients with histologically diagnosed MASLD for training and validation studies; for the MASLD and MASH with fibrosis groups, specificity and sensitivity were 80–90%, with an AUC of approximately 0.9.

Our study underscores the pivotal role of FF as a primary biomarker in diagnosing MASH among liver transplant recipients. Employing machine learning techniques, particularly decision tree analysis, alongside MR methods, blood biomarkers, and clinical parameters, we achieved high specificity and sensitivity in both training and validation cohorts. Notably, secondary variables like insulinemia and elastography further refined the classification process, highlighting the efficacy of a comprehensive, data‐driven approach in MASH diagnosis.

### 4.1 Limitations of the Study

The primary limitation of our study is the small cohort size, which may affect certain machine‐learning applications. Additionally, the similar character of our liver transplant patient cohort, resulting from standardized treatment protocols, may limit the applicability of our findings to the general population, where greater variability in clinical parameters exists.

Finally, while MR spectroscopy, used to calculate FF, requires expertise, it can be substituted with MR imaging proton density fat fraction, accessible on most commercial imagers.

## 5 Conclusion

Our analysis demonstrates that MR‐based fat fraction (FF) measurements serve as a robust imaging biomarker for hepatic steatosis in the general population and effectively differentiate between non‐steatosis, MASLD, and MASH in liver transplant recipients, achieving high sensitivity and specificity (AUC > 0.96). The positive predictive value (PPV) for diagnosing MASH post‐transplantation ranged from 90% to 97% using decision‐tree models and approximately 88% using ROC analysis—significantly higher than the general population (PPV 45%–78%). Secondary biomarkers, such as insulinemia and elastography, enhanced classification accuracy, particularly near the FF threshold of 5.3%. These findings underscore the clinical value and precision of MR spectroscopy as a noninvasive biomarker for MASH diagnosis in transplant patients.

## Conflicts of Interest

The authors declare no conflicts of interest.

## Data Availability

The datasets generated or analyzed during the study are not publicly available due to ethical reasons but are available from the corresponding author on reasonable request.
